# Nanomedicine-based lactate metabolism and lactylation regulation for exploring new therapeutic strategies in cancer

**DOI:** 10.1016/j.mtbio.2026.102893

**Published:** 2026-02-05

**Authors:** Shuzhe Cai, Siqi Li, Jingjing Liu

**Affiliations:** a School of Traditional Chinese Medicine, Faculty of Medicine, Yangzhou University, Yangzhou, 225009, China; b Key Laboratory of the Jiangsu Higher Education Institutions for Nucleic Acid & Cell Fate Regulation (Yangzhou University), Yangzhou, 225009, China

**Keywords:** Functional nanomaterials, Lactate metabolism, Protein lactylation, Tumor microenvironment modulation, Catalytic therapy

## Abstract

Lactate, a key metabolite of glycolysis in tumor cells, plays a dual role in cancer progression. On one hand, it contributes to the formation of an acidic tumor microenvironment that fosters tumorigenesis and malignancy. On the other, it promotes tumor progression through protein lactylation, a recently identified post-translational modification. Consequently, targeting lactate metabolism and lactylation represents a critical therapeutic method in oncology. Advances in nanomedicine have opened new possibilities for precise modulation of lactate metabolism. Nanomedicine-based strategies enable the regulation of lactate production, transport, and clearance, thereby offering effective tools for tumor suppression. Additionally, the lactate induced lactylation orchestrated by lactyl-CoA, lactyltransferases, and delactylases offer more refined targets for investigating approaches to inhibit tumor progression. In this review, we comprehensively summarize recent developments in nanomedicine-based strategies for lactate metabolism modulation and the mechanism of lactylation regulation. We suppose the design of multifunctional nanoplatforms capable of simultaneously regulating lactate levels and lactylation could integrate metabolic and epigenetic interventions to disrupt lactate-driven tumor support mechanisms precisely and effectively for advancing cancer treatment paradigms.

## Graphical abstract

**Figure:**
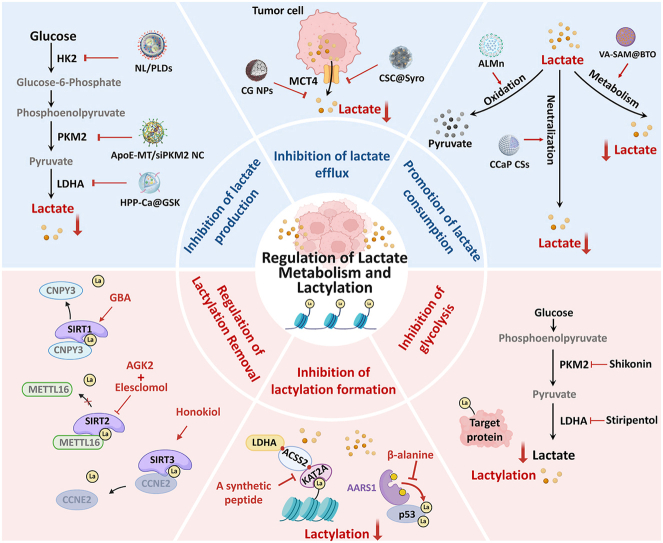

## 1 Introduction

**Fig. 1:**
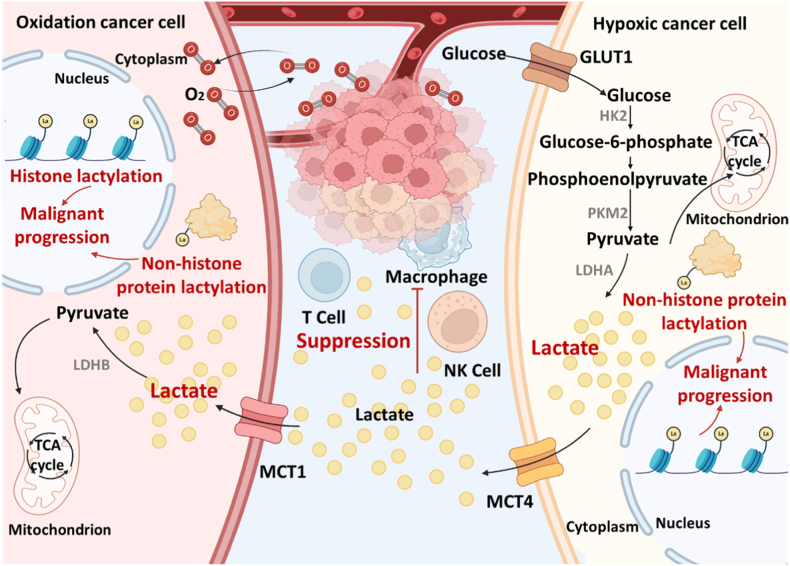
Lactate metabolism and protein lactylation in tumor cells are intimately associated with malignant tumor progression. Created in https://Biorender.com.

Although current cancer treatment modalities, including surgery, chemotherapy, radiotherapy, and immunotherapy, have achieved substantial progress [1], the adaptive evolution of tumor cells, particularly their metabolic reprogramming, which leads to the accumulation of various metabolic byproducts within the tumor microenvironment (TME), ultimately promotes tumor progression and therapeutic resistance [2]. Among these byproducts, lactate has emerged as one of the most critical barriers to effective cancer treatment [3,4]. Malignant tumor cells survive via the Warburg effect, preferentially relying on glycolysis over mitochondrial oxidative phosphorylation (OXPHOS) for energy production, even under normoxic conditions [5]. During glycolysis in cancer cells, aberrant activation of key enzymes such as hexokinase 2 (HK2), pyruvate kinase M2 (PKM2), and lactate dehydrogenase A (LDHA) drives excessive lactate production. To prevent intracellular acidification, lactate is exported via monocarboxylate transporter 4 (MCT4), thereby maintaining intracellular pH homeostasis (Fig. 1) [6]. The accumulation of lactate in the extracellular area acidifies the TME, which in turn alters immune cell function and impairs the efficacy of surgery, chemotherapy, radiotherapy, and immunotherapy [[7], [8], [9]].

Given the detrimental impact on cancer therapy, lactate metabolism has garnered increasing attention as a therapeutic target. It has been found that inhibition of key glycolytic enzymes in tumor cells could reduce lactate production [10], while blocking MCT4 and MCT1 could suppress lactate shuttle [11]. Notably, 2-deoxy-D-glucose (2-DG) has been tested in early clinical trials (NCT00096707) to inhibit key glycolytic enzyme HK2 for the purpose of regulating lactate production and suppressing tumor growth, but its efficacy was limited, likely due to poor selectivity and dose-limiting toxicity [12]. Additionally, the MCT1 inhibitor AZD3965 is the first and currently the only drug that has entered a phase I clinical trial (NCT01791595), providing direct clinical evidence for the feasibility of targeting lactate shuttling in cancer patients [13]. Additionally, the administration of lactate oxidase (LOX) promotes lactate degradation [[14], [15], [16]]. However, conventional strategies targeting lactate metabolism with small-molecule inhibitors are limited by poor specificity and solubility, constraining their clinical application [17]. Recent advances in nanomedicine offer approaches to precisely modulate lactate metabolism within the TME [18]. Various nanoscale carriers were developed to deliver therapeutic agents more effectively to tumors with improved drug stability, increased tumor accumulation, and reduced off-target effects, making them well suited for drug-based metabolic intervention enhancement [19]. These nanoplatforms not only facilitate the direct delivery of small-molecule inhibitors to modulate lactate levels within the TME but also enable co-administration with other therapeutic agents for synergistic cancer treatment [20]. In certain designs, the nanoparticles can even directly neutralize lactate by themselves [21,22]. These nanomedicine-based approaches markedly enhance drug delivery efficiency and bioavailability, offering a promising strategy to target tumor lactate metabolism [23].

Notwithstanding these advancements, most current strategies mainly focus on lowering lactate levels or blocking its transport in the TME, while the comprehensive biological functions of lactate remain inadequately explored. Growing data indicates that lactate transcends its role as a mere metabolic waste and can actively influence tumor progression through epigenetic mechanisms [24]. Therefore, strategies that solely regulate lactate metabolism may be insufficient, necessitating a more profound comprehension of lactate-driven molecular regulation. Lactate, often considered solely a metabolic waste, is now recognized as a significant signaling and epigenetic regulator. Lactate can function as a donor for protein lactylation, a recently discovered post-translational modification that influences both histone and non-histone proteins [25]. Histone lactylation promotes oncogenic transcriptional programs, while non-histone lactylation frequently regulates proteins linked to malignant progression, altering their function, and modulating responses to therapy [26]. Moreover, protein lactylation in conjunction with other oncogenic proteins can establish a positive feedback loop that amplifies metabolic reprogramming [27,28]. Therefore, regulating protein lactylation is of great importance for improving tumor therapy.

Early attempts to inhibit lactylation mainly focused on reducing lactate production by suppressing glycolytic enzymes. With the investigation of lactylation mechanism, it has been found that lactate promotes protein lactylation through both lactyl-CoA-dependent and independent pathways [29]. Despite the markedly low concentration of lactyl-CoA in human cells, significantly lower than that of acetyl-CoA, cancer cells may employ succinyl-CoA synthetase and acyl-CoA synthetase to operate as lactyl-CoA synthetases, facilitating the efficient production of lactyl-CoA and subsequent lactylation. Moreover, the elevated lactate concentration in the tumor may enhance its affinity for alanyl-tRNA synthetase, which acts as a lactate sensor and facilitates global lysine lactylation in tumor cells independent of lactyl-CoA. Therefore, blocking critical enzymes in either pathway can reduce lactylation and hinder malignant tumor development [29]. Conversely, delactylation, catalyzed by specific delactylases, can reverse this modification, and restrain tumor development. Thus, the regulation of delactylases provides an alternative approach to modulate lactylation [30]. However, current lactylation regulation approaches predominantly rely on inhibitors [31], which often suffer from poor tumor specificity and limited bioavailability. This highlights an urgent need to develop targeted nanoplatforms to enhance delivery efficiency and therapeutic efficacy.

**Fig. 2:**
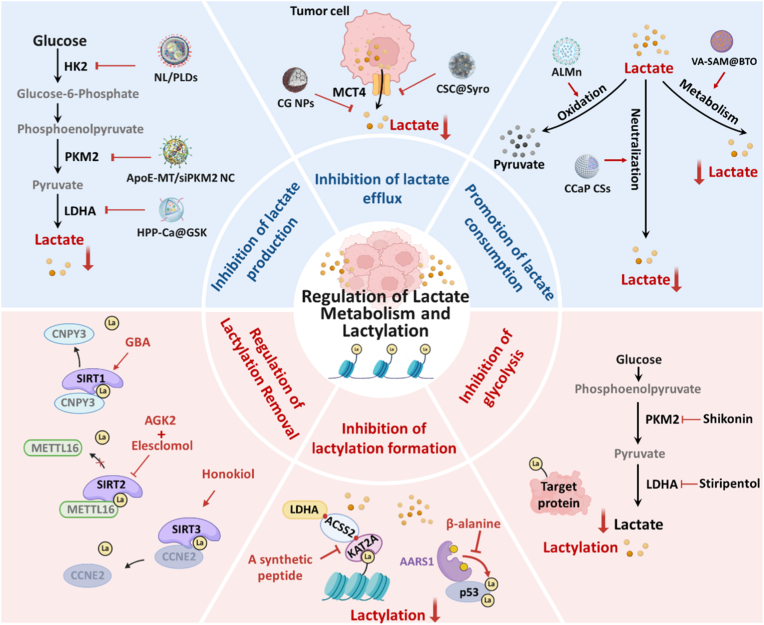
Strategies for targeting tumor-associated lactate metabolism and protein lactylation. Created in https://Biorender.com.

This review aims to provide a comprehensive overview of recent advances in nanomedicine-enabled regulation of lactate metabolism in tumors (Fig. 2) and emerging approaches and mechanisms for modulating protein lactylation, highlighting current challenges and opportunities. Based on these insights, we propose a forward-looking strategy: the design of multifunctional nanoplatforms capable of simultaneously regulating lactate levels and lactylation modifications. This integrated metabolic-epigenetic intervention strategy offers a precise and synergistic approach to tumor treatment, potentially ushering in a new paradigm in cancer therapy.

## 2 Nanomedicine-based strategies for modulating lactate metabolism

The metabolic reprogramming of cancer cells to preferentially engage in glycolysis for energy production even under normoxic conditions is driven by the aberrant upregulation of glycolytic enzymes such as HK2, PKM2, and LDHA, resulting in elevated lactate and adenosine triphosphate (ATP) synthesis [32,33]. To counteract the threat of intracellular acidification, lactate is transported out of the cell by MCT4, preserving cytosolic pH. However, the persistent export of lactate leads to acidification of the TME, which fosters tumor adaptation, immune resistance, neovascularization, and metastatic progression [34].

**Table 1:** Summary of nanomedicine-based strategies for inhibiting lactate production in tumor cells.

Strategies	Specific Method	Directly inhibitory components of lactate	Mechanism	Ref.
Inhibition of Lactate Production	Inhibition of HK2	LND	Inhibiting HK2 to suppress glycolysis, reducing lactate production, and mitigating CD8^+^ T cell impairment to potentiate immunotherapy.	Zhang et al. [35]
		3BP	Deactivating HK2 to block lactate production and reprograming TAMs to an M1-like phenotype. Combines with PTT.	Yang et al. [36]
		2-DG	Inhibiting HK2 to suppress lactate production, remodeling immunosuppressive TME (suppress M2 macrophages, downregulate Tregs).	Dai et al. [37]
		LND	Inhibiting HK2-mediated glycolysis and disrupting mitochondrial OXPHOS to overcome cisplatin resistance.	Lu et al. [38]
	Inhibition of PK	siPKM2	Inhibiting lactate production, disrupting energy supply, and overcoming TMZ resistance.	Zhang et al. [39]
		Serine	Promoting PKM2 tetramerization to reverse aberrant dimer activation, inhibiting tumor proliferation and resistance.	Wang et al. [40]
	Inhibition of LDH	GSK	Inhibiting LDHA to suppress aerobic glycolysis, shifting metabolism to high glucose/low lactate, and potentiating sonodynamic therapy & immunotherapy.	Yan et al. [41]
		CRISPR/Cas9 RNP	Reprograming cellular metabolism, enhancing cuproptosis and anti-tumor immunity.	Luan et al. [42]
Inhibition of lactate efflux	Inhibition of MCT4	Syro	Blocking lactate efflux, enhancing photothermal therapy.	Yang et al. [43]
		Galactose moieties (drug-free)	Blocking lactate efflux, reversing immunosuppressive TME through intracellular lactate accumulation.	Hsu et al. [44]
Promotion of lactate consumption	Oxidation of Lactate by LOX	LOX	Enhancing catalytic therapy and reversing the immunosuppressive TME to potentiate anti-tumor immunity.	Liu et al. [45]
		LOX	Normalizing tumor vasculature and depleting lactate to reverse immunosuppression, thereby significantly augmenting ICB therapy.	Chen et al. [46]
	Neutralization of lactate by the nanomaterials	Sodium Bicarbonate (NaHCO_3_)	Reversing immunosuppression and inducing pyroptosis, thereby amplifying antitumor immunity and enhancing immunotherapy efficacy.	Ding et al. [47]
		Disodium Hydrogen Phosphate	Reversing the immunosuppressive tumor microenvironment and synergizing with pyroptosis induction to amplify antitumor immunity and enhance immunotherapy efficacy.	Ding et al. [48]
		Calcium Carbonate (CaCO_3_)	Reversing the immunosuppressive tumor microenvironment and synergizing with ICD induction to potentiate immunotherapy efficacy.	Fang et al. [49]
		CaCO_3_	Reversing the immunosuppressive tumor microenvironment, enhancing immunogenic cell death, and potentiating immunotherapy by promoting T-cell infiltration and activation.	Zheng et al. [21]
		CaCO_3_	Reversing the immunosuppressive TME by alleviating acidity and hypoxia, thereby enhancing the efficacy of both immune checkpoint blockade (anti-PD-1) and CAR-T cell immunotherapy.	Dong et al. [22]
	Engineered Bacteria for Lactate Metabolism	*VA*	Enhancing T cell infiltration and dendritic cell maturation, and potentiating immunotherapy by remodeling the metabolic and immune microenvironment of colorectal cancer.	Fan et al. [50]
		TPZ	Promoting dendritic cell maturation, increasing cytotoxic T-cell infiltration, and reducing regulatory T-cell activity, thereby synergizing with immune checkpoint blockade and CAR-T cell therapies.	Li et al. [51]

Despite conventional small-molecule inhibitors have been utilized for lactate metabolism, their clinical translation remains limited due to challenges such as poor aqueous solubility and insufficient selectivity [17]. Inspired by the development of nanomedicine, nanoplatform-mediated delivery of inhibitors targeting HK2, PKM2, or LDHA can effectively disrupt lactate biosynthesis [20]. Furthermore, MCT4-specific nanoplatforms can block lactate export pathways [11], whereas LOX-loaded nanoformulations can catalyze lactate degradation, thereby mitigating immunosuppression and restraining tumor growth [[14], [15], [16]]. These strategies improve pharmacokinetic profiles and therapeutic specificity of small-molecule inhibitors and enzymes, highlighting the potential of nanomedicine in targeting interference of lactate-driven oncogenic metabolism (Table 1).

### 2.1 Inhibition of lactate production

The hyperactive glycolysis in tumor cells is initiated by HK2, which catalyzes the phosphorylation of glucose to yield glucose-6-phosphate. Then the glucose-6-phosphate undergoes the intermediate reactions of glycolysis, culminating in the generation of phosphoenolpyruvate, which is subsequently transformed to pyruvate by PKM2. Ultimately, LDHA reduces pyruvate to lactate [52]. These cascade enzyme-catalyzed reactions contribute to the lactate production within tumors. Thus, regulating these enzymes provides a direct strategy to inhibit glycolytic lactate generation at its source in the TME.

#### 2.1.1 Inhibition of HK2

HK2 is overexpressed in various cancers, catalyzing glucose phosphorylation to accelerate glycolysis and lactate production [53]. Its overexpression is tightly associated with tumor aggressiveness, fueling both the metabolic demands of rapid cancer cell proliferation and the development of cancer stemness, chemoresistance, and immune evasion, which are the features linked to poor clinical outcomes [54,55]. Up to date, lots of chemicals such as 3-bromopyruvate (3BP), lonidamine (LND), and 2-deoxy-D-glucose (2-DG) have been developed to inhibit the function of HK2 for cancer treatment.

**Fig. 3:**
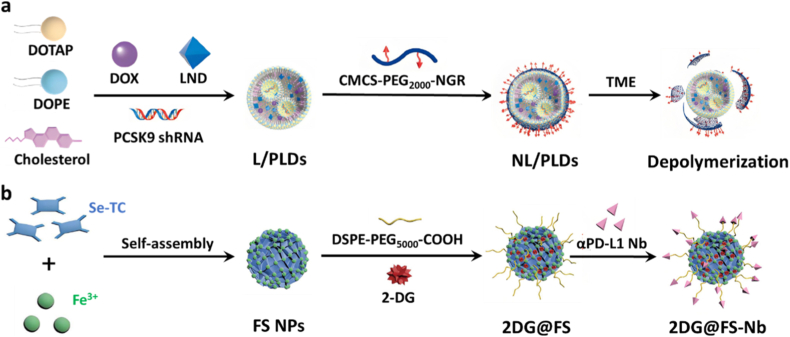
(a) Schematic illustration of the synthesis of NL/PLDs [35]. Copyright 2025, Elsevier. (b) Schematic representation of 2DG@FS-Nb fabrication [37]. Copyright 2024, John Wiley and Sons.

Zhang et al. developed an innovative lipid nanoparticle system through thin-film dispersion and extrusion methods, realizing the co-encapsulation of a PCSK9-targeting shRNA plasmid, the HK2 inhibitor LND, and low-dose doxorubicin (DOX) within the core of nanoparticles (L/PLDs) (Fig. 3a). These nanoparticles were subsequently functionalized with polymer consisting of pH-responsive carboxymethyl chitosan (CMCS), polyethylene glycol (PEG), and tumor-targeting NGR peptides to generate the final product (NL/PLDs). Zeta potential measurements further demonstrated that NL/PLDs exhibited a distinct pH-dependent surface charge conversion. Under physiological conditions, the nanoparticles maintained a negative surface charge, whereas under mildly acidic conditions, corresponding to the tumor microenvironment, the surface charge shifted toward positive values. This pH-responsive charge reversal provides a physicochemical basis for the efficient dissociation of the CMCS shell and the subsequent accelerated release of DOX, PCSK9 shRNA, and LND in acidic environments. Upon encountering the acidic TME, the CMCS underwent charge reversal from negative to positive, which not only triggered the polymer dissociation from the lipid core but also facilitated rapid internalization of cationic L/PLDs by tumor cells. Intracellular payload release of DOX stimulated T-cell activation via immunogenic cell death (ICD) induction, while PCSK9 shRNA restored MHC-I-mediated antigen presentation. Critically, LND suppressed HK2-mediated glycolysis at its source, curtailing tumor lactate production and thereby mitigating lactate-induced impairment of CD8^+^ T cell effector function. In murine melanoma models, NL/PLDs combined with anti-PD-1 therapy resulted in significant tumor volume reduction and markedly elevated intratumoral CD8^+^ T cell infiltration, demonstrating the role of HK2-targeted lactate modulation in potentiating cancer immunotherapy [35].

In addition to immunological potentiation, HK2 inhibitors synergistically enhance antitumor activity when used with other treatment methods. Yang et al. developed a biomimetic nanodrug platform by encapsulating 3BP-loaded mesoporous Prussian blue nanoparticles (MPB NPs) within genetically engineered programmable cell membranes with expression of a variant of signal-regulatory protein α (SIRPα) [36]. The released 3BP, as a glycolytic inhibitor, deactivates HK2 and blocks lactate production, while simultaneously reprogramming tumor-associated macrophages (TAMs) toward an M1-like antitumor phenotype. MPB NPs also serve as photothermal sensitizers, enabling photothermal therapy (PTT)-induced tumor ablation. Additionally, by competitively blocking the CD47-SIRPα "don't eat me" interaction through its binding to CRC cells, the SIRPα variant dual-functionally enables both precise tumor targeting and efficient macrophage-mediated phagocytosis. This triple-combination strategy based on MPB-3BP@CM NPs containing photothermal ablation, metabolic rewiring, and immune remodeling exerts potent synergistic effects on tumor suppression. When combined with PTT, the HK2 inhibitors could also downregulate regulatory T cell levels apart from regulating the TAM phenotype transformation. Dai et al. synthesized a D-A-D-type NIR-II molecule (Se-TC) as a photothermal agent and then the Se-TC was coordinated with Fe^3+^ to form the nanoparticle through self-assembly. After loading with 2-DG and further surface conjugation of anti-PD-L1 nanobodies (Nb), the nanoplatform 2DG@FS-Nb was generated for enhanced immunotherapy by tumor metabolism reprogramming (Fig. 3b) [37]. The formation of uniformly dispersed nanoparticles resulting from Fe^3+^-coordinated self-assembly was confirmed by TEM, while elemental mapping verified the homogeneous distribution of Fe within the nanostructure. DLS analysis further demonstrated a narrow nanoscale size distribution and excellent colloidal stability after Nb conjugation, which supports efficient tumor accumulation. This nano system delivers 2-DG to inhibit HK2, thereby suppressing lactate production to remodel the immunosuppressive TME by suppressing M2 macrophage proliferation and downregulating regulatory T cell levels. The surface-conjugated Nb enables precise immune blockade at tumor lesions. Upon 1064 nm laser irradiation, 2DG@FS-Nb exhibited rapid photothermal conversion, with the temperature increasing to 67.4 °C, and its strong NIR-II optical absorption also resulted in a markedly enhanced photoacoustic signal under 1064 nm excitation. In this system, Se-TC could not only be used as a photothermal agent but also serve as a NIR-II imaging contrast agent for real-time, high-resolution localization of deep-seated tumors. Spectroscopic characterization confirmed strong NIR-II optical absorption and emission, enabling simultaneous imaging and photothermal therapy. In the meanwhile, the Fe^3+^ in the 2DG@FS-Nb could consume GSH to enhance the Fe^2+^-based Fenton reaction for elevated ferroptosis. Thus, this antibody-guided “see-and-treat” modality introduces a new paradigm for immunometabolism therapy, transitioning lactate-targeting strategies into a precision-guided, image-assisted therapeutic era.

Beyond immune modulation, HK2 inhibitors can also overcome chemoresistance. Cisplatin (CDDP), a widely used chemotherapeutic agent, is often limited by resistance and adverse effects. To address this, Lu et al. designed a GSH-responsive nanoplatform by conjugating the glycolytic inhibitor LND to the cisplatin prodrug (Pt (IV)) via a disulfide bond, further decorated with TPP, to form a multifunctional prodrug molecule termed LND-SS-Pt-TPP (LPT). This prodrug was subsequently encapsulated using β-cyclodextrin-modified hyaluronic acid (HA-CD), leading to the self-assembly of nanoparticles designated as LPT/HA-CD NPs. Among LPT/HA-CD, TPP refers to triphenylphosphine, which targets mitochondria, while HA-CD targets the tumor CD44 receptor. Thus, this nanoplatform showed dual targeting synergistically efficacy. LND not only inhibits HK2-mediated glycolysis but also disrupts mitochondrial OXPHOS, sensitizing cancer cells to cisplatin. *In vivo* studies confirmed that LPT/HA-CD effectively restored chemosensitivity in cisplatin-resistant lung cancer cells [38], offering a new strategy to combat drug resistance.

#### 2.1.2 Inhibition of PK

Pyruvate kinase (PK) is a critical enzyme catalyzing that catalyzes the final step of glycolysis in tumor cells by converting phosphoenolpyruvate to pyruvate with the generation of ATP. Mammalian PK comprises four isoforms including PKM1, PKM2, PKR, and PKL. Among them, PKM2 is markedly upregulated in various tumors, which promotes glycolytic flux and the resulting formation of lactate, both of which have a substantial impact on the results of treatment [56]. Consequently, inhibiting tumor PKM2 represents a promising strategy for cancer treatment [57].

**Fig. 4:**
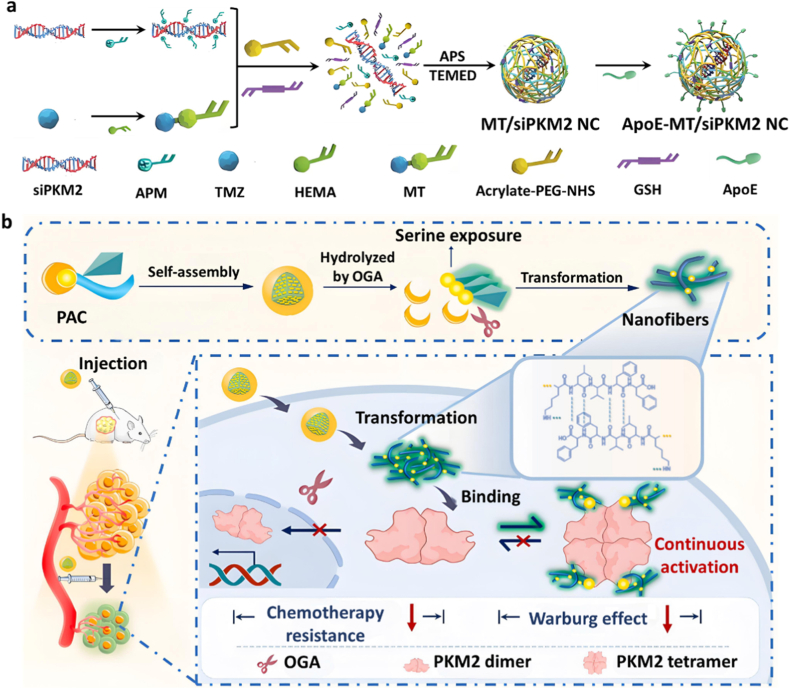
(a) Schematic illustration of the synthesis of ApoE-MT/siPKM2 nano capsules (NCs) [39]. Copyright 2024, John Wiley and Sons. (b) Mechanistic diagram of the *in vivo* function and assembly of the PKM2 allosteric converter (PAC) [40]. Copyright 2023, Elsevier.

Research has indicated that glioblastoma multiforme (GBM) exhibits overexpression of PKM2, which is closely linked to resistance to temozolomide (TMZ), a leading chemotherapy drug. Zhang et al. developed a TMZ nano capsule (ApoE-MT/siPKM2 NC) by encapsulating siRNA targeting PKM2 (siPKM2) as the core, with a polymer shell composed of methacrylate-TMZ (MT) and glutathione (GSH)-responsive cross linker with disulfide bonds and acrylate-PEG2K-NHS after ammonium persulfate (APS) and N, N, N′,N′-Tetramethyl ethylenediamine (TEMED) initiate a free radical polymerization reaction. Thereafter, the surface of nano capsules was modified with apolipoprotein E (ApoE) peptides to realize BBB penetration and GBM targeting (Fig. 4a) [39]. Given the intrinsic instability of siRNA and its susceptibility to RNase-mediated degradation, agarose gel electrophoresis was employed to evaluate the protective capability of the nanocapsules. The results showed that naked siRNA was rapidly degraded in the presence of RNase, whereas siRNA encapsulated within ApoE-MT/siPKM2 NC remained largely intact even after prolonged incubation, indicating that the nanocapsule structure effectively protected siRNA from enzymatic degradation and enabled its successful intracellular delivery. In the high-GSH environment of GBM, the structure of nano capsules decomposed and then released MT and siPKM2. *In vitro* experiments showed that ApoE-MT/siPKM2 NC significantly inhibited lactate production in U87 cells by suppressing PKM2 expression and decreased intracellular ATP levels to disrupt energy supply, thereby alleviating TMZ resistance and promoting tumor cell apoptosis.

PKM2 is mostly found in tumor cells as interconverting tetramers and dimers, and the PKM2 dimers translocate to the nucleus to regulate gene transcription associated with tumor proliferation, metastasis, and chemoresistance [58]. Therefore, apart from directly inhibiting the expression of PKM2, maintaining PKM2 in its tetrameric form is also a crucial approach for effective tumor therapy. Serine, a natural PKM2 ligand, activates tetramerization, but it is rapidly metabolized and cannot sustain allosteric modulation. To overcome this limitation, Wang et al. developed a PKM2 allosteric converter (PAC), which comprises three functional modules: an enzyme-cleavable β-N-acetylglucosamine (GlcNAc)-protected serine site, a self-assembling peptide (KLVFF), and an aggregation-induced emission (AIE) motif [40]. Upon PAC nanoparticles reaching the tumor site, overexpressed O-GlcNAcase (OGA) cleaves the GlcNAc group, triggering self-assembly of peptides into nanofibers with exposed serine residues. These nanofibers sustain local serine release, stabilizing PKM2 tetramers and effectively reversing aberrant PKM2 dimers activation. *In vivo* studies demonstrated that co-administration of PAC with sunitinib significantly reduced tumor burden in mice and effectively overcame sunitinib resistance in renal cell carcinoma (RCC) (Fig. 4b)

#### 2.1.3 Inhibition of LDH

Lactate dehydrogenase (LDH) is a tetrameric isoenzyme consisting of three subunits, LDHA, LDHB, and LDHC, which generate five isoforms (LDH1-5) that catalyze the reversible conversion between lactate and pyruvate in glycolysis [59]. Among these, LDHA specifically catalyzes the reduction of pyruvate to lactate and serves as a driver of the Warburg effect. Clinical studies have demonstrated that LDHA is overexpressed in various cancers, with its elevated levels significantly correlating with tumor malignancy and poor prognosis [60,61].

**Fig. 5:**
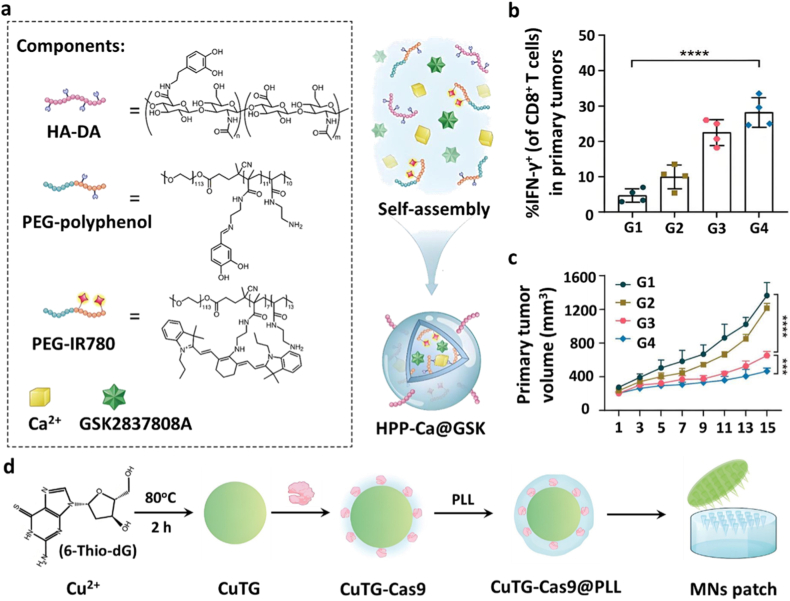
(a) Schematic illustration of the composition of HPP-Ca@GSK nanoparticles [41]. Copyright 2023, American Chemical Society. (b) Quantification of IFN-γ levels in tumor tissues under various treatment conditions(G1:PBS, G2:aCLAT4, G3:HPP-Ca@GSK + US, G4: HPP-Ca@GSK + US + aCLAT4) [41]. Copyright 2023, American Chemical Society. (c) Tumor volume measurements in mice subjected to different treatments [41]. Copyright 2023, American Chemical Society. (d) Schematic diagram of the synthesis of CuTG-Cas9@PLL nanohybrids [42]. Copyright 2024, John Wiley and Sons.

Several LDHA inhibitors have been identified to date, including FX11 [62], GSK2837808A (GSK) [63], and Stiripentol [64]. To enhance their delivery efficiency and therapeutic efficacy, a range of nanodrug delivery systems has been developed for targeted delivery of these LDHA inhibitors. For example, Yan et al. engineered a functionalized metal-phenolic network nanoparticle for efficient GSK loading and LDHA inhibition. In this system, the GSK was incorporated into a nanostructure via coordination-driven self-assembly of hyaluronic acid-dopamine (HA-DA), PEG-modified polyphenols, PEG-conjugated photosensitizer IR780, and calcium chloride (CaCl_2_), forming HPP-Ca@GSK (Fig. 5a). Hyaluronic acid facilitated active targeting of tumor cells with high CD44 expression, enabling selective uptake and precise intracellular delivery of GSK for suppressed aerobic glycolysis in cancer cells, leading to a metabolic shift characterized by high glucose and low lactate levels. This reprogrammed TME enhanced CD8^+^ T cell cytotoxicity and disrupted Treg cell stability, thereby potentiating antitumor immune responses. Stability assays demonstrated that the coordination of calcium with polyphenolic groups provided structural stability to the nanoparticles while enabling ultrasound-responsive release. As the ultrasound exposure time increased, the nanoparticles generated enhanced ROS. Simultaneously, ultrasound-induced oxidative stress significantly enhanced the calcium overload and impaired mitochondrial function, triggering the release of damage-associated molecular patterns (DAMPs) for dendritic cell (DC) maturation and subsequent activation of CD8^+^ T cells. Due to the destabilization of Treg cells by anti-CTLA-4 (aCTLA-4) therapy, tumors treated with HPP-Ca@GSK + US + aCTLA-4 exhibited enhanced infiltration of CD8^+^ T cells secreting proinflammatory cytokines, resulting in significant tumor regression in 4T1-bearing mice compared to other groups (Fig. 5b and c) [41].

Beyond delivering LDHA inhibitors, cutting-edge technologies such as CRISPR/Cas9 gene editing were also developed to knockout the LDHA gene, enabling precise regulation of lactate production. Luan et al. constructed a three-dimensional network nanostructure by coordinating 6-thio-2′-deoxyguanosine (6-Thio-dG) with Cu^2+^ via sulfur-metal bonds (CuTG). TEM revealed that CuTG formed an interconnected network-like nanostructure, confirming successful sulfur–Cu^2+^ coordination-driven assembly. Negatively charged CRISPR/Cas9 ribonucleoproteins (RNPs) were electrostatically adsorbed onto the CuTG surface and subsequently coated with bovine serum albumin (BSA) and poly-L-lysine (PLL), forming CuTG-Cas9@PLL nanohybrids. Through vacuum-assisted molding, the CuTG-Cas9@PLL nanohybrids were incorporated into dissolvable microneedle patches using sulfobutylether-β-cyclodextrin (SCD) as a matrix, enabling transdermal drug delivery for melanoma treatment (Fig. 5d). Upon internalization by tumor cells, the Cas9 and 6-Thio-dG from CuTG-Cas9@PLL could enhance the Cu^2+^-induced cuproptosis by ablating the LDHA to reprogram cellular metabolism and inducing telomeric stress to boost anti-tumor immunity, respectively [42].

### 2.2 Inhibition of lactate shuttle

The Monocarboxylate Transporter (MCT) family comprises 14 members that facilitate proton-coupled lactate transport across cell membranes, critically regulating intracellular pH homeostasis and metabolic crosstalk in tumors. Among these, MCT4 is notably overexpressed in hypoxic tumor cells [65]. MCT4 facilitates lactate efflux across the plasma membrane, maintaining intracellular pH homeostasis and supporting key malignant phenotypes such as proliferation, migration, invasion, angiogenesis, and immune evasion [66]. Blocking MCT4 stops lactate from leaving hypoxic tumor cells, causing lactate to build up inside, which leads to acidity and stress. In the meanwhile, the glycolytic substrates of oxygenated tumor cells would be deprived, leading to the disrupted energy metabolism and cell death. Furthermore, during this process, MCT4 inhibition remodels the acidic TME, thereby enhancing antitumor immune responses [11]. Thus, MCT4 inhibition offers a promising strategy to potentiate immunotherapy via metabolic reprogramming. Additionally, MCT1 is highly expressed in aerobic tumor cells with the function of mediating the uptake of extracellular lactate and providing fuel for OXPHOS, making it a key player in maintaining tumor metabolic symbiosis [67,68].

**Fig. 6:**
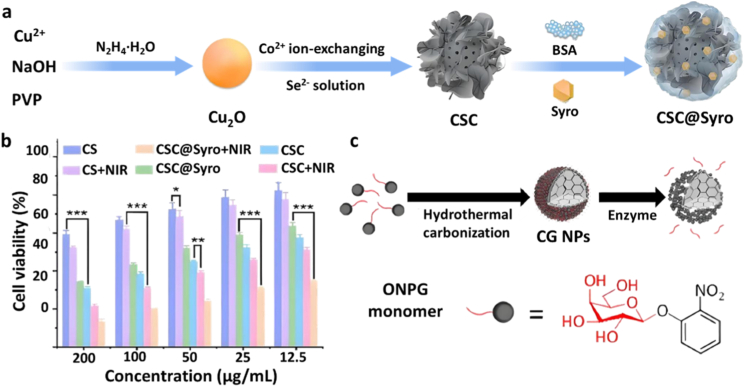
(a) Schematic illustration of the synthesis of CSC@Syro nanoparticles [43]. Copyright 2023, American Chemical Society. (b) Cell viability of 4T1 cells following various treatments [43]. Copyright 2023, American Chemical Society. (c) Schematic representation of the synthesis of CG nanoparticles (CG NPs) [44]. Copyright 2024, American Chemical Society.

Several MCT4 inhibitors, including quercetin, syrosingopine (Syro) [69], and fluvastatin [70], have shown preclinical promise in cancer and cardiovascular disease treatment, however, their clinical translation is hindered by poor bioavailability and off-target effects. To overcome these problems, Yang et al. developed a stimuli-responsive nanocarrier system for Syro loading. First, utilizing Cu_2_O as a precursor, monodisperse hollow CuSe nanospheres (CS NPs) were synthesized via the Kirkendall diffusion effect. Then, the CuSe/CoSe_2_ heterostructured nanoparticles (CSC NPs) were formed through Co^2+^ ion exchange and Se^2−^ doping. The hollow interior of the CuSe nanospheres and the successful formation of CuSe/CoSe_2_ heterostructures were confirmed by TEM, while elemental mapping verified the uniform distribution of Cu, Co, and Se within the CSC nanoparticles. Surface modification with BSA enabled hydrophobic loading of the MCT4 inhibitor Syro, forming the CSC@Syro nanocomposite (Fig. 6a). Drug loading studies demonstrated efficient Syro encapsulation within the BSA-modified CSC nanoparticles, and NIR-triggered release assays confirmed accelerated Syro release upon 808 nm irradiation. Owing to the presence of Cu^+^/Cu^2+^ and Co^2+^/Co^3+^ redox couples, CSC nanoparticles catalyze the conversion of hydrogen peroxide (H_2_O_2_) into highly reactive hydroxyl radicals (•OH) via a Fenton-like reaction, thereby directly inducing ferroptosis in tumor cells. Simultaneously, the generated Cu^2+^ and Co^3+^ species deplete intracellular GSH, establishing a self-amplifying redox cycle that further enhances oxidative stress and reinforces the ferroptosis cascade. Upon 808 nm near-infrared (NIR) irradiation, the photothermal effect of the CSC heterostructure triggers localized hyperthermia, loosening the BSA shell and enhancing molecular vibrations, thus promoting rapid Syro release. Released Syro inhibits lactate efflux from tumor cells into the TME, causing intracellular acidification and alleviating TME acidity. The resulting intracellular acid toxicity synergizes with ferroptosis to enhance ICD, while lowered lactate levels relieve acid-mediated immunosuppression, thereby augmenting immunotherapy efficacy (Fig. 6b) [43].

Apart from small-molecule inhibitor, Hsu's team innovated a drug-free biomimetic nanoplatform by designing glycosylated nanoparticles that mimic MCT4 inhibitor functionality. Specifically, carbonized glyconanoparticles (CG NPs) enriched with galactose moieties on their surface were fabricated via hydrothermal carbonization of o-nitrophenyl-β-galactosidase (ONPG) precursors (Fig. 6c). CG NPs enter GL261 glioma cells via galactose receptor-mediated endocytosis. Furthermore, the surface-exposed galactose moieties of CG NPs could competitively bind to glycosylation sites on MCT4, thereby blocking its lactate export function and reversing the immunosuppressive state of the TME [44]. Concurrently, the accumulation of intracellular lactate induces acid toxicity, which synergizes with immune checkpoint blockade therapy to potentiate antitumor immunity.

For MCT1 inhibition, Chen and colleagues developed a core-shell nanoplatform by using a zirconium-based porphyrin metal-organic framework (PZM) as the core for MCT1 inhibitor α-cyano-4-Hydroxycinnamate (CHC) loading and then coated it with hyaluronic acid (HA) as the shell for tumor cell CD44-targeted delivery (CHC-PZM@HA). The nanoparticles release CHC inside tumor cells, inhibiting MCT1-mediated lactate uptake, forcing the tumor cells to switch from lactate-fueled oxidative respiration to glycolysis, significantly reducing oxygen consumption and reversing the tumor hypoxic microenvironment. This endogenous oxygenation strategy effectively enhanced the singlet oxygen yield of the porphyrin ligand in PZM under light exposure, ultimately improving the photodynamic therapy effect for tumors [71].

### 2.3 Promotion of lactate consumption

Due to enhanced glycolytic activity, lactate concentrations in tumor tissues are significantly elevated (10-40 mM), markedly exceeding those in normal tissues (0.1-1 mM) [10]. The efflux of lactate from tumor cells leads to the formation of an acidic TME, which in turn promotes malignant phenotypes. Beyond strategies targeting lactate production or transport inhibition, facilitating intratumoral lactate consumption has also emerged as an effective approach to remodel the immunosuppressive microenvironment and enhance therapeutic efficacy. Current methodologies developed to promote lactate consumption within tumor cells primarily fall into three categories: enzymatic degradation using LOX, chemical neutralization via functional materials, and microbial metabolism employing engineered bacteria.

#### 2.3.1 Oxidation of lactate by LOX

LOX, a natural flavin mononucleotide (FMN)-dependent enzyme produced by certain bacteria, catalyzes the oxidation of lactate into pyruvate and H_2_O_2_ in the presence of oxygen [72]. To improve the bioavailability and tumor-specific accumulation of LOX, various nanocarriers such as metal-organic frameworks (MOFs) [73], cationic liposomes [74], and hydrogels [75] have been developed for targeted delivery. However, the catalytic efficiency of LOX is oxygen-dependent, and its activity is severely limited in the hypoxic TME [12]. To address this, Zhang et al. constructed a metal-phenolic network-based nanocomposite that co-delivers LOX and atovaquone (ATO), a mitochondrial complex III inhibitor [76]. ATO suppresses mitochondrial oxygen consumption, thereby alleviating tumor hypoxia and improving LOX activity. Nonetheless, the efficacy of this approach remains suboptimal in the context of intrinsically hypoxic solid tumors [72].

**Fig. 7:**
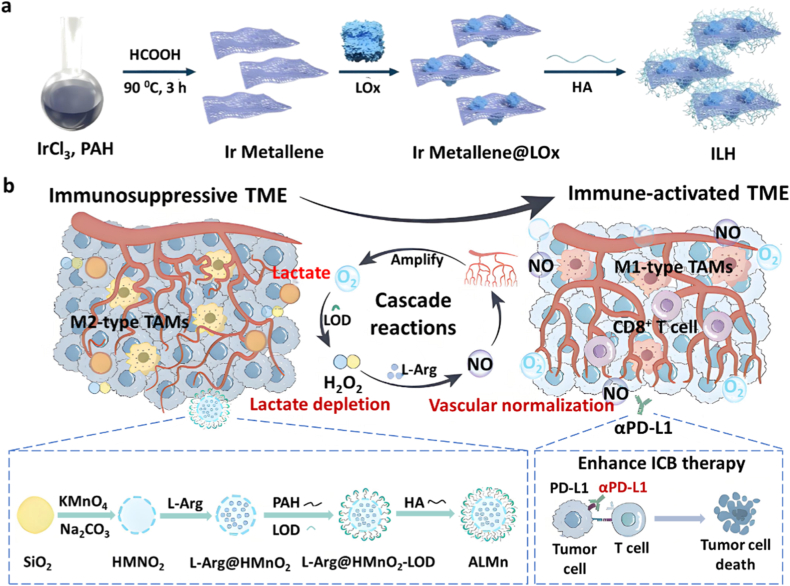
(a) Schematic illustration of the synthesis of the dual-enzyme cascade reaction platform (ILH) [45]. Copyright 2024, American Chemical Society. (b) Schematic representation of the synthesis of ALMn nanoparticles [46]. Copyright 2025, Elsevier.

To further overcome oxygen limitations, dual-enzyme engineered nano systems capable of simultaneously delivering LOX and oxygen-generating nanozymes *in situ* were developed. For instance, Liu et al. synthesized an iridium-based nanozyme via wet chemical reduction with excellent biocompatibility and peroxidase-like catalytic activity [45]. Then, LOX was immobilized on the nanozyme surface by electrostatic adsorption, followed by HA modification to achieve tumor targeting, resulting in the construction of a dual-enzyme cascade nanoplatform termed ILH (Fig. 7a). Furthermore, ILH exhibits exceptional photothermal performance that accelerates lactate oxidation by LOX, while simultaneously being utilized for photoacoustic (PA) imaging to monitor PA signal variations resulting from oxygen-induced changes in oxygenated hemoglobin (OxyHb) and deoxygenated hemoglobin (DeoxyHb) concentrations. This integrated approach enables real-time dynamic tracking of the cascade catalytic therapy process. *In vitro* assays demonstrated that ILH effectively reduced lactate levels and increased intratumoral oxygen concentrations, thereby amplifying LOX-mediated metabolic remodeling.

Given that the H_2_O_2_ generated from LOX-catalyzed lactate oxidation can further react with L-arginine (L-Arg) to produce nitric oxide (NO), promoting vascular normalization and alleviating immune suppression, Chen et al. encapsulated L-Arg into hollow manganese dioxide (HMnO_2_) nanoparticles to form L-Arg@HMnO_2_, subsequently coated the surface with poly(allylamine hydrochloride) (PAH) via layer-by-layer assembly, electrostatically adsorbed LOX, and stabilized it with HA to generate the ALMn nanoplatform (Fig. 7b). Monitoring of lactate concentration revealed that ALMn consistently consumed lactate while promoting H_2_O_2_ production, indicating that the nanoreactor retained the catalytic activity of LOX. Subsequently, H_2_O_2_ oxidized L-Arg to produce large amounts of NO, significantly higher than when H_2_O_2_ and L-Arg were provided separately. This system exhibited potent lactate-oxidizing capacity, and vascular permeability assays confirmed that ALMn significantly enhanced tumor vascular normalization [46].

#### 2.3.2 Neutralization of lactate by the nanomaterials

**Fig. 8:**
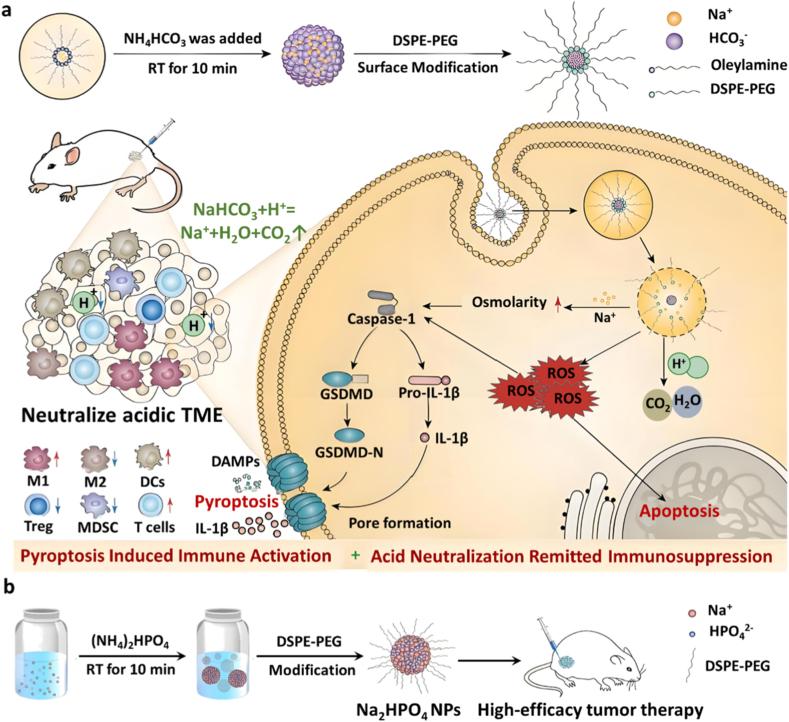
(a) Schematic illustration of the synthesis of NaHCO_3_ nanoparticles (NaHCO_3_ NPs) and their therapeutic mechanism [47]. Copyright 2023, John Wiley and Sons. (b) Schematic illustration of the synthesis of disodium hydrogen phosphate nanoparticles (Na_2_HPO_4_ NPs) [48]. Copyright 2025, John Wiley and Sons.

Chemical neutralization of lactate using alkaline nanomaterials has emerged as a promising approach to reverse the acidic TME [77,78]. NaHCO_3_, a clinically approved agent for systemic and tissue pH regulation, has been evaluated in both preclinical and clinical cancer settings. However, excessive systemic administration may induce metabolic alkalosis and impair respiratory compensation, underscoring the need for tumor-targeted delivery systems. To address this challenge, Ding et al. developed NaHCO_3_ nanoparticles (NaHCO_3_ NPs) using a rapid microemulsion method at room temperature, employing sodium oleate and ammonium bicarbonate as precursors. Surface modification with DSPE-PEG significantly improved particle dispersion and biocompatibility (Fig. 8a). The NaHCO_3_ NPs exhibited a uniform spherical morphology with an average diameter of 70 nm, as observed in TEM images. High-resolution TEM further revealed a lattice spacing of 0.24 nm corresponding to the (211) plane of crystalline NaHCO_3_. X-ray diffraction patterns confirmed the formation of the pure NaHCO_3_ phase, and FTIR spectra verified the successful surface modification with DSPE-PEG. Upon reaching the tumor site, the nanoparticles could neutralize lactate by releasing bicarbonate ions (HCO_3_^−^) via acid-base reactions, thereby modulating lactate metabolism. Ion-release studies demonstrated rapid and sustained Na^+^ release under acidic conditions, enabling NaHCO_3_ NPs to function as intracellular ion reservoirs. *In vitro*, NaHCO_3_ NPs demonstrated superior pH buffering in 4T1 breast cancer media compared to free bicarbonate [47]. Moreover, the high level of sodium ions released by NaHCO_3_ NPs increased intracellular osmotic pressure, triggering pyroptosis and ICD, hence promoting the release of DAMPs and pro-inflammatory cytokines to enhance antitumor immune responses. Similarly, this team prepared disodium hydrogen phosphate nanoparticles (Na_2_HPO_4_ NPs) via microemulsion (Fig. 8b), demonstrating superior lactate-neutralizing efficacy compared to free Na_2_HPO_4_ in 4T1 tumor-bearing mice, as well as inducing tumor suppression through pyroptosis [48].

**Fig. 9:**
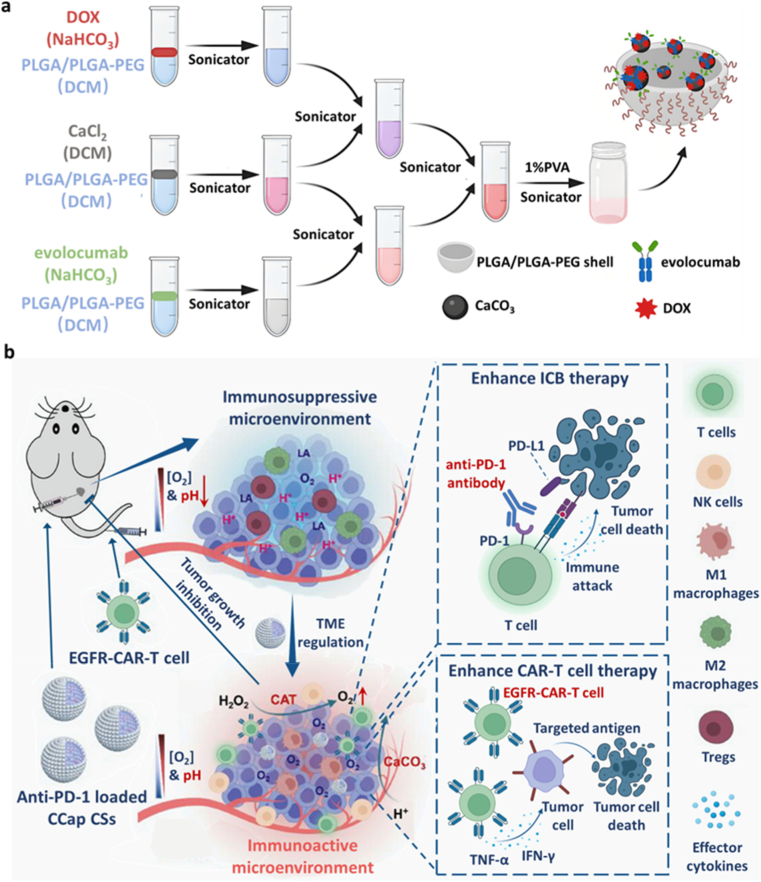
(a) Schematic illustration of the synthesis of DECP nanoparticles [49]. Copyright 2024, American Chemical Society. (b) CCaP CSs enhance tumor ICB therapy and CAR-T cell therapy by modulating the TME [22]. Copyright 2023, John Wiley and Sons.

Furthermore, CaCO_3_ with lactate neutralization ability offers a multifunctional platform due to its low solubility, sustained HCO_3_^−^ release profile, drug-loading capacity [79]. For instance, Fang et al. employed a CaCO_3_-assisted double emulsion technique to co-load DOX and the PCSK9-targeting monoclonal antibody, evolocumab, into PLGA-PEG nanoparticles, generating DOX/evolocumab@CaCO_3_-PLGA-PEG (DECP) (Fig. 9a). The spherical morphology of DECP nanoparticles was confirmed by TEM, and pH-responsive dissolution assays showed that CaCO_3_ effectively neutralized acidity under TME-mimicking conditions. After TME acidity neutralization by CaCO_3_, the immunosuppressive microenvironment was reshaped, and DOX uptake was also increased by increasing membrane permeability, thereby promoting ICD. By blocking PCSK9, evolocumab enhances antigen presentation and boosts CD8^+^ T cell infiltration. Thus, when combined with PD-L1 blockade [49], DECP showed an amplified synergistic effect of chemotherapy and immunotherapy.

In addition to bicarbonate buffering, calcium ions released from CaCO_3_ decomposition also play a crucial role in tumor therapy. Zheng et al. encapsulated DOX and erianin into CaCO_3_-based nanoparticles (DECaNPs) via double emulsion. CaCO_3_ not only neutralized lactate to enhance immunotherapy but also released Ca^2+^ to induce calcium overload. Additionally, the erianin released from DECaNPs could upregulate L-type calcium channels to further facilitate Ca^2+^ influx to induce mitochondrial apoptosis and following tumor cell death [21]. Thus, DECaNPs could realize synergistically enhanced chemoimmunotherapy via increased calcium overload and acidity neutralization. Apart from loading chemotherapeutics and evolocumab into nanosized CaCO_3_, Dong et al. developed microsized CaCO_3_ colloidosomes assembled by hydrophobic nanosized CaCO_3_ with catalase (CAT) and anti-PD-1 antibodies encapsulated through the double emulsion method(CCaP CSs). Laser confocal microscopy with different fluorescent markers directly confirmed that CAT and anti-PD-1 antibodies were successfully encapsulated within the shell composed of CaCO_3_ and PLGA. Due to the presence of CaCO_3_, the nanoparticles effectively neutralized the acidic environment, leading to an increase in the pH of the solution. In this triple-combination strategy, CaCO_3_ neutralizes intratumoral lactate, CAT decomposes H_2_O_2_ into O_2_ to alleviate hypoxia, and anti-PD-1 restores immune surveillance. This approach enhanced chimeric antigen receptor T-Cell (CAR-T) efficacy, achieving complete tumor remission in 80% of mice [22] (Fig. 9b).

#### 2.3.3 Metabolism of lactate by engineered bacteria

Despite the promising regulatory potential of nanomaterial-based lactic acid neutralization strategies, their catalytic efficiency is fundamentally limited by insufficient tumor penetration depth, heterogeneous intratumoral distribution, and pH/enzyme-dependent activity loss in the dynamic TME [18]. In recent years, several natural microorganisms such as *Veillonella atypica* (*VA*) [80], *Shewanella oneidensis* [81], *Eubacterium hallii* [82], and *Gluconobacter oxydans* [83] have been identified with intrinsic capabilities to metabolize lactic acid. Leveraging the native metabolic functions of live bacteria, these organisms exhibit superior *in vivo* stability and higher catalytic efficiency compared to synthetic nanomaterials, shifting the paradigm from “static neutralization” to “dynamic regulation” of lactate metabolism [84]. More importantly, due to their unique respiratory and metabolic profiles, many anaerobic bacteria exhibit inherent tumor tropism under hypoxic conditions, enabling active penetration into deep tumor regions. This allows for localized accumulation and enhancement of antitumor activity [[85], [86], [87]].

**Fig. 10:**
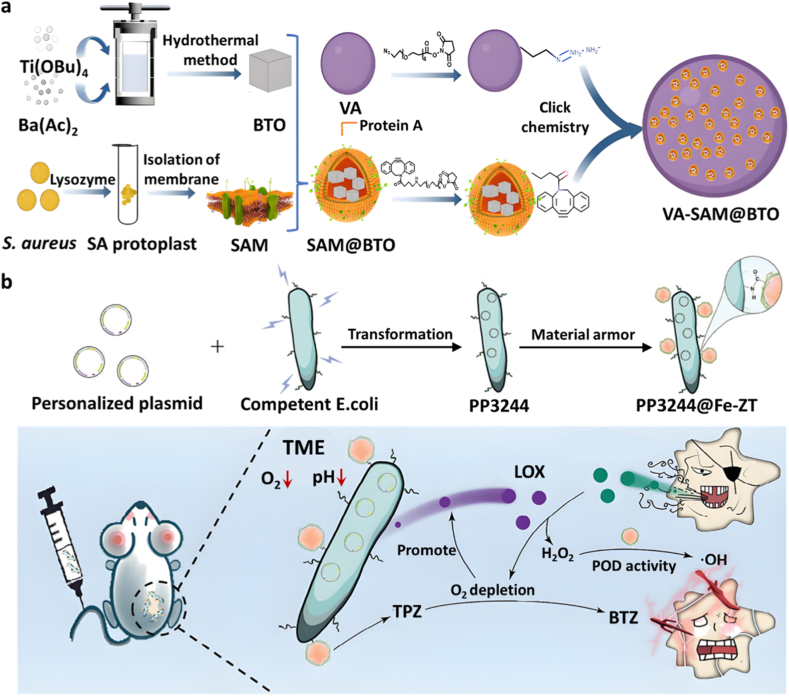
(a) Schematic illustration of the synthesis of the VA-SAM@BTO microrobot. From Ref. [50] © The Authors, some rights reserved; exclusive licensee AAAS. Distributed under a CC BY-NC 4.0 license http://creativecommons.org/licenses/by-nc/4.0/”. (b) Schematic representation of the construction of engineered PP3244@Fe-ZT bacteria and there *in vivo* therapeutic process [51]. Copyright 2024, John Wiley and Sons.

Fan et al. synthesized piezoelectric barium titanate (BaTiO_3_, BTO) nanocubes via a hydrothermal method and coated them with a *Staphylococcus aureus* membrane (SAM). These SAM-coated BTO nanocubes (SAM@BTO) were then conjugated onto the surface of VA to generate a biologically hybrid microrobot, VA-SAM@BTO, through click reaction (Fig. 10a). The perovskite crystal structure of BTO nanocubes was confirmed by X-ray diffraction, and TEM revealed their well-defined cubic morphology. Successful SAM coating was further verified by the retention of membrane proteins and changes in surface charge. This hybrid microrobot could actively target tumor tissues by inflammatory targeted-SAM and hypoxic targeted-VA. Upon sonication, the BTO nanocubes on the bacterial surface can generate large quantities of reactive oxygen species (ROS) and carbon monoxide (CO) to synergistically induce ICD and catalyze the oxidative coupling of VA cells metabolized lactate, effectively disrupting the immunosuppressive microenvironment. In an orthotopic murine colorectal cancer model, oral administration of VA-SAM@BTO combined with ultrasound exposure significantly reduced tumor volume owing to the synergetic catalysis and immunotherapy [50].

**Fig. 11:**
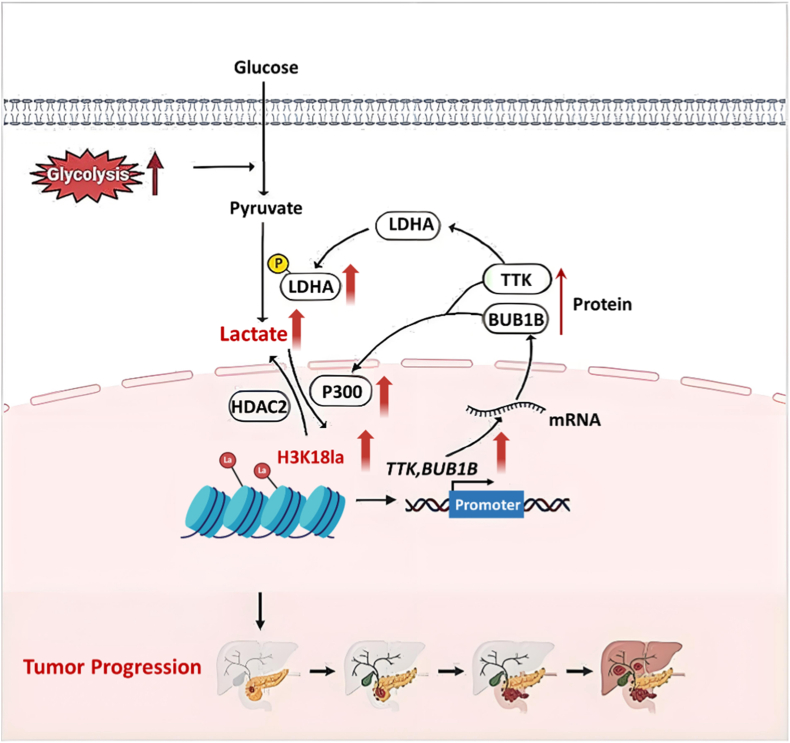
Schematic illustration of LDHA-induced H3K18 lactylation in PDAC, where LDHA interacts with TTK and BUB1B to establish a positive feedback loop [27]. Copyright 2024, Springer Nature.

However, the clinical application of microorganisms faces significant challenges due to their nonspecific metabolic activity, generation of toxic byproducts, and uncontrolled proliferation [88,89]. Intravenous administration of live microbes has been associated with severe complications, including cytokine storms and sepsis [90,91]. To date, only a few microbial products have received approval for clinical trials [92]. Therefore, it is critical to engineer probiotic strains that can safely colonize tumor tissues while minimizing systemic risks. Li et al. developed a hybrid *Escherichia coli*-based therapeutic platform combining genetic engineering and nanomaterial functionalization for TME-responsive targeting and improved biosafety (Fig. 10b). Hypoxia-inducible LOX expression in engineered *E. coli* was validated at both transcriptional and protein levels, confirming oxygen-dependent metabolic activation. Meanwhile, TEM and elemental mapping verified the successful deposition of Fe-functionalized the Zeolitic imidazolate framework-8 (ZIF-8) nanoparticles on the bacterial surface. Elemental mapping further demonstrated the successful deposition of the nanoparticles on the bacterial surface. Additionally, the engineered bacteria maintained their efficient lactate consumption ability. Hypoxia-inducible expression of LOX in engineered *E. coli* (PP3244) enabled lactate depletion, H_2_O_2_ generation and O_2_ depletion. In the meanwhile, the ZIF-8 nanoparticles encapsulating tirapazamine (TPZ) were synthesized and further functionalized with Fe^2+^ to yield Fe-ZT, exhibiting pH-sensitive TPZ release with enhanced therapeutic effect under hypoxia and peroxidase-like activity for generation •OH. *In vivo* experiments demonstrated that systemic administration of PP3244@Fe-ZT significantly reduced lactate levels within tumor tissues and upregulated the expression of co-stimulatory molecules CD80 and CD86 on DCs, indicating enhanced antigen-presenting capacity [51].

## 3 Inhibition of protein lactylation

Although nanomedicine offers methods to modulate tumor lactate metabolism for enhancing anti-cancer treatment, growing evidence suggests that lactate is much more than a metabolic byproduct [93]. Beyond contributing to an immunosuppressive microenvironment, lactate also acts as a precursor for protein lactylation [25], an epigenetic modification known to activate the transcription of oncogenes [94,95]. Consequently, numerous tactics have been devised to prevent protein lactylation, including the suppression of glycolysis to reduce lactate levels and the meticulous regulation of lactylation formation and removal by the modulation of individual enzymes associated with different lactylation pathways (Table 2).

**Table 2:** Summary of strategies for regulating tumor lactylation.

Strategies	Specific Method	Directly inhibitory components of lactate	Mechanism	Ref.
Inhibition of Glycolysis	Inhibition of LDHA	Stiripentol	Inhibits LDHA to reduce lactate production, thereby decreasing H3K9 lactylation and overcoming TMZ resistance.	Chen et al. []
	Inhibition of PKM2	Shikonin	Inhibits PKM2 to suppress lactate production and subsequent AIM2 lactylation, sensitizing cells to ferroptosis.	Yue et al. [96]
		D34-919	Disrupts PKM2 tetramerization to reduce lactate generation and XRCC1 lactylation, overcoming therapy resistance.	Li et al. [97]
Inhibition of Lactylation Formation	ACSS2-KAT2A Complex	Synthetic Peptide	Competitively inhibits the lactyltransferase complex, suppressing histone lactylation (H3K14la, H3K18la) and tumor immune evasion.	Zhu et al. [98]
	AARS1/AARS2	β-Alanine	Competitively binds to AARS1, inhibiting its lactyltransferase activity and preventing p53 lactylation to enhance chemotherapy.	Zong et al. [99]
Enhancing the Removal of Lactylation Modifications	SIRT1	GBA	Activates SIRT1 delactylase activity, promoting CNPY3 delactylation to induce tumor cell pyroptosis.	Zhang et al. [100]
	SIRT3	HKL	Activates SIRT3 to catalyze CCNE2 delactylation, inhibiting tumor growth.	Jin et al. [101]
	SIRT2	AGK2 + Elesclomol	Inhibits SIRT2 delactylase activity to increase METTL16 lactylation, promoting cuproptosis. Combined with copper ionophore for synergy.	Huang et al. [102]

### 3.1 Inhibition of glycolysis

Given that glycolysis serves as the primary process of lactate production, efforts to modulate protein lactylation in tumors naturally centered on inhibiting key glycolytic enzymes to limit lactate production, thereby inhibiting lactylation and its downstream oncogenic signaling. Enzymes such as LDHA and PKM2, commonly overexpressed in tumor tissues, have emerged as therapeutic targets, as their inhibition could restrain lactate production and disrupt metabolic-epigenetic coupling.

LDHA is a key enzyme that promotes protein lactylation in tumor cells. Li et al. found that in pancreatic ductal adenocarcinoma (PDAC), LDHA overexpression enhances lactate production, leading to histone H3K18 lactylation. This modification upregulates TTK protein kinase (TTK) expression, which in turn promotes the phosphorylation and enzymatic activity of LDHA, perpetuating a positive feedback loop (Fig. 11) [27]. Furthermore, elevated lactate levels induced by LDHA could substantially elevate H3K9 lactylation in the LUC7L2 promoter to initiate LUC7L2 transcription, enhancing its expression. LUC7L2 facilitates the retention of intron 7 in MLH1, resulting in diminished MLH1 expression and impaired mismatch repair (MMR), which eventually contributes to resistance to TMZ. Stiripentol, a clinically approved antiepileptic drug, has been shown to overcome TMZ resistance in glioblastoma by inhibiting LDHA/B-mediated histone H3K9 lactylation [].

It has been reported that PKM2 overexpression could also induce histone lactylation, contributing to the treatment resistance. Recently, Li et al. discovered that in glioblastoma, the interaction between aldehyde dehydrogenase 1 family member A3 (ALDH1A3) and PKM2 facilitates the tetramerization of PKM2, leading to increased lactate accumulation and the lactylation of X-ray repair cross complementing protein 1 (XRCC1) at lysine 247 (K247). Lactylated XRCC1 exhibits an increased affinity for importin α, facilitating better nuclear translocation of XRCC1 and improved DNA repair mechanisms [97]. A high-throughput screen identified compound D34-919, which could enhance the sensitivity of cancer cells to chemoradiotherapy by disrupting the ALDH1A3-PKM2 interaction and reducing lactate generation and XRCC1 lactylation.

### 3.2 Inhibition of lactylation formation

Although protein lactylation could be regulated by inhibiting the activity of glycolytic enzymes such as LDHA and PKM2, the process and mechanism of lactylation regulation still need to be clearly elucidated. Detailly, protein lactylation can occur via two distinct pathways: a lactyl-CoA-dependent pathway and a lactyl-CoA-independent pathway [29]. In lactyl-CoA-dependent pathway, lactyl-CoA synthetases catalyze the conjugation of lactate and CoA to form lactyl-CoA, which serves as a direct chemical donor of the lactyl group to transfer the lactyl moiety to lysine residues on target proteins through specific lactyltransferases catalysis [98,103]. In the lactyl-CoA-independent pathway, intracellular lactate-sensing proteins could serve as a bona fide lactyltransferase that can directly use lactate and ATP to catalyze protein lactylation [28,99]. Understanding the regulatory mechanisms underlying both pathways and targeting the key enzymes involved in each can effectively suppress the formation of protein lactylation.

#### 3.2.1 Inhibition of the lactyl-CoA-dependent pathway

**Fig. 12:**
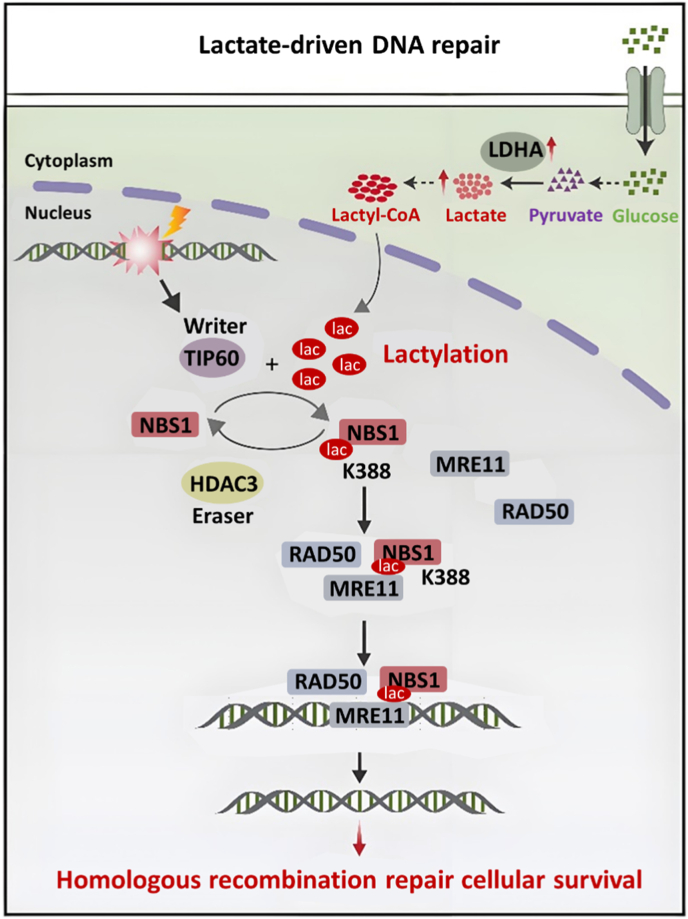
Schematic illustration showing that, in cisplatin-resistant gastric cancer cells, LDHA induces NBS1 lactylation, thereby promoting radio- and chemoresistance [105]. Copyright 2024, Springer Nature.

In the lactyl-CoA-dependent pathway, lactyltransferases recognize and use lactyl-CoA derived from lactate to transfer lactyl groups onto lysine residues of target proteins. This modification connects cellular metabolism with epigenetic regulation and drives oncogenic programs [104]. Chen et al. demonstrated that lactate-induced lactylation of nijmegen breakage syndrome protein 1 (NBS1) occurs with TIP60 acting as a potential lysine lactyltransferase to accept lactyl-CoA in the cofactor pocket for the formation of the RAD50-MRE11-NBS1 (MRN) complex. The MRN complex could enhance homologous recombination repair in tumor cells and impair the efficacy of chemotherapy and radiotherapy (Fig. 12). In cisplatin-resistant gastric cancer cells, lactylation at lysine 388 of the NBS1 protein could be selectively inhibited by stiripentol. Stiripentol limits lactyl-CoA availability by reducing the lactate production, leading to the decreased lactylation of NBS1 at K388. Consequently, MRN complex formation and homologous recombination repair are attenuated, enhancing tumor sensitivity to cisplatin-induced DNA damage. When used in combination with cisplatin, stiripentol markedly suppressed tumor growth and prolonged survival in mouse models [105]. Given the side effect of the MRN complex in cancer treatment, Chen's group designed a specific cell-penetrating peptide (K673-pe) to target and inhibit lactylation at site K673 of the MRE11 in the MRN complex for weakened binding to DNA and then suppressed the homologous recombination (HR) repair process. In preclinical studies, including patient-derived xenograft (PDX) models, the combination of K673-pe with the PARP inhibitor olaparib or cisplatin significantly enhanced the cytotoxic effects of chemotherapy and effectively reversed tumor chemoresistance mediated by MRE11 lactylation [106].

**Fig. 13:**
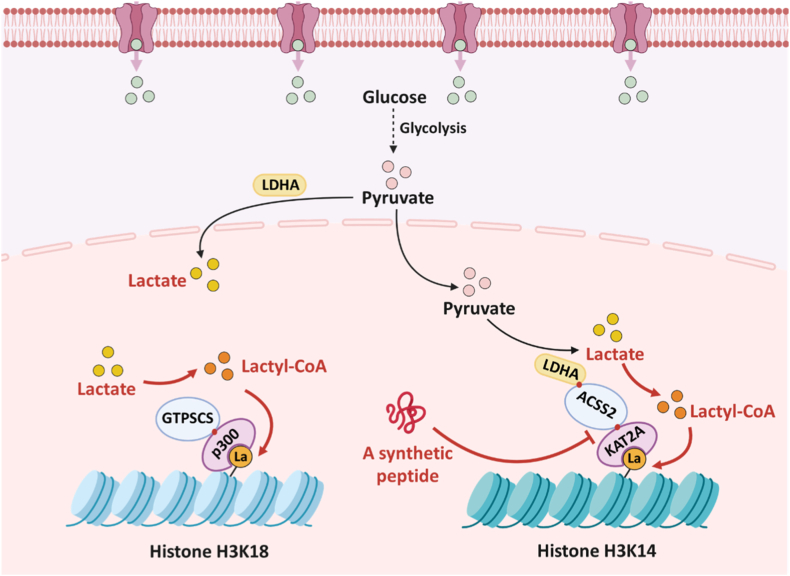
Schematic illustration of how the GTPSCS-ACSS2 complexes promote histone lactylation in the lactyl-CoA-dependent pathway, along with corresponding regulatory strategies. Created in https://Biorender.com.

However, the level of lactyl-CoA in human cells is extremely low, far below that of acetyl-CoA. Thus, cancer cells utilize guanosine 5′-triphosphate (GTP)-specific succinyl-CoA synthetase (GTPSCS) and acyl-CoA synthetase short-chain family member 2 (ACSS2) to function as lactyl-CoA synthetases for efficient generation of lactyl-CoA (Fig. 13).

Although GTPSCS was originally known as a mitochondrial enzyme that catalyzes succinyl-CoA formation [107,108], it was later found to localize in the nucleus and use GTP to convert lactate into lactyl-CoA [103]. In the nucleus, GTPSCS interacts directly with the lactyltransferase p300, forming a functional complex to catalyze protein lactylation. The lactyl-CoA synthesized by GTPSCS is then efficiently transferred to the nearby catalytic center of p300 to drive histone H3K18 lactylation [103]. This complex makes sure that even when global lactyl-CoA levels are low, local levels are still enough to support.

The phosphorylation and nuclear translocation of ACSS2, a nuclear acetyl-CoA synthetase, were promoted by the activation of the EGFR-ERK-mediated pathway. In the nucleus, the ACSS2 forms a complex with lysine acetyltransferase 2A (KAT2A) [98]. LDHA produces lactate, ACSS2 converts it to lactyl-CoA, and KAT2A transfers the lactyl group to histones. This complex also greatly increases the catalytic efficiency of protein lactylation, thereby promoting tumor progression. Based on this mechanism, a synthetic peptide was developed to block the ACSS2-KAT2A interaction competitively. This inhibition can precisely reduce H3K14la and H3K18la, leading to decreased tumor cell proliferation and immune evasion. *In vivo*, this peptide markedly enhances the efficacy of PD-1-based immunotherapy by lowering PD-L1 expression and relieving T-cell exclusion through inhibition of ACSS2-KAT2A-driven H3K14la and H3K18la [98].

#### 3.2.2 Inhibition of the lactyl-CoA-independent pathway

Alanyl-tRNA synthetase 1 (AARS1) and alanyl-tRNA synthetase 2 (AARS2) are best known for catalyzing the attachment of alanine to tRNA, a core step in protein synthesis [109,110]. Under normal conditions, owing to the low lactate levels (1.5-3.0 mM) and the weak affinity of lactate for AARS1/2, these enzymes mainly perform their canonical functions, making lactylation inefficient. However, in pathological states such as tumors, the high lactate concentration increases its affinity against AARS1/2 and promotes lactylation even if the intrinsic catalytic efficiency of AARS1/2 for lactate may remain lower than for alanine. Zhou et al. found that AARS1 and AARS2 can act as a lactate sensor and lactyltransferase, directly binding to lactate and ATP to form lactyl-AMP, subsequently transferring the activated lactyl moiety from lactyl-AMP to lysine residues of target proteins [99]. As a result, AARS1/2 can drive global protein lactylation in tumor cells, promoting malignant progression.

**Fig. 14:**
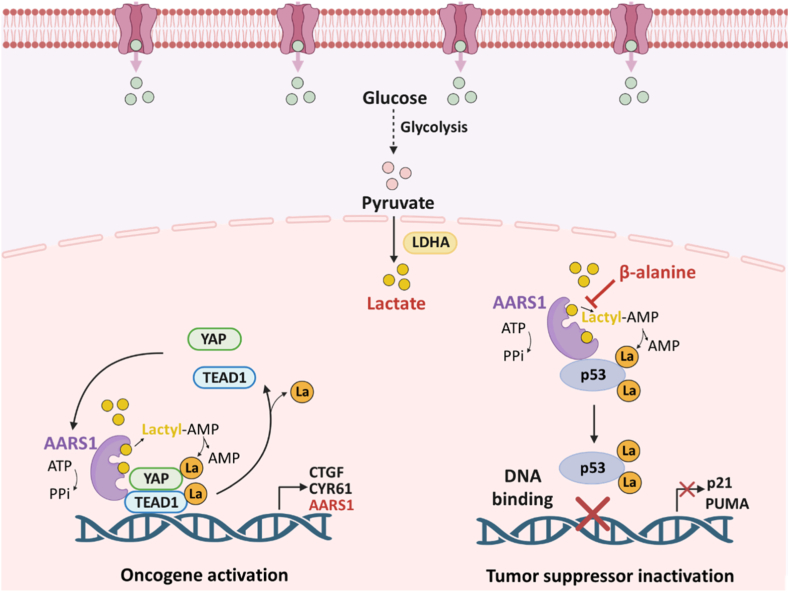
Schematic illustration of AARS1-catalyzed non-histone lactylation in the lactyl-CoA-independent pathway, together with its regulatory strategies. Created in https://Biorender.com.

AARS1 primarily acts in the nucleus to drive tumor progression (Fig. 14). In gastric cancer, AARS1 catalyzes the lactylation of the transcription factors Yes-associated protein (YAP) and TEA domain family member 1 (TEAD1), thereby promoting oncogene activation. Notably, AARS1 itself is one of the genes upregulated by the YAP-TEAD1 complex. Thus, the positive feedback loop between AARS1 and the YAP-TEAD1 complex lactylation continuously accelerates tumor progression [28]. Additionally, AARS1 also directly promotes lactylation of dual lysine residues on the tumor suppressor protein p53, leading to impaired DNA binding, weakened liquid-liquid phase separation, and reduced transcriptional activity. Consequently, p53 inactivation downregulates downstream genes such as p21 and p53 upregulated modulator of apoptosis (PUMA), thereby facilitating tumor progression. Notably, β-alanine can competitively bind AARS1 and inhibit p53 lactylation, enhancing chemotherapy. In animal models, β-alanine markedly reduced tumor growth and improved survival [99]. Moreover, AARS2 mainly functions in mitochondria and could mediate lactylation of cyclic GMP-AMP synthase (cGAS), suppressing innate immune signaling and promoting immune evasion [111].

### 3.3 Regulation of lactylation removal

Tumor cells promote protein lactylation through both lactyl-CoA-dependent and -independent pathways, reshaping protein functions and gene expression. However, lactylation is not a static or irreversible modification [94,112]. It has been demonstrated that some enzymes can recognize and remove lactyl groups in proteins, establishing a dynamic balance of lactylation levels [113,114]. This reversible balance opens new possibilities for precisely regulating tumor lactylation and enhancing therapeutic responses.

Current evidence indicates that classical class I histone deacetylase (HDAC) and members of the Sirtuin family can act as “moonlighting” delactylases [115]. Although the lactyl group is bulkier than the acetyl group, the catalytic pocket of delactylases can still accommodate and hydrolyze the lactyl group attached to lysine residues [116]. Class I HDACs, including HDAC1, HDAC2, and HDAC3, can effectively remove histone lactylation at sites such as H3K18la in tumor cells [115]. Additionally, inhibition or knockdown of HDAC1/2/3, using specific class I HDAC inhibitors such as entinostat, leads to a marked accumulation of histone lactylation [115]. These findings suggest that class I HDACs serve as key regulators maintaining the dynamic balance of histone lactylation.

**Fig. 15:**
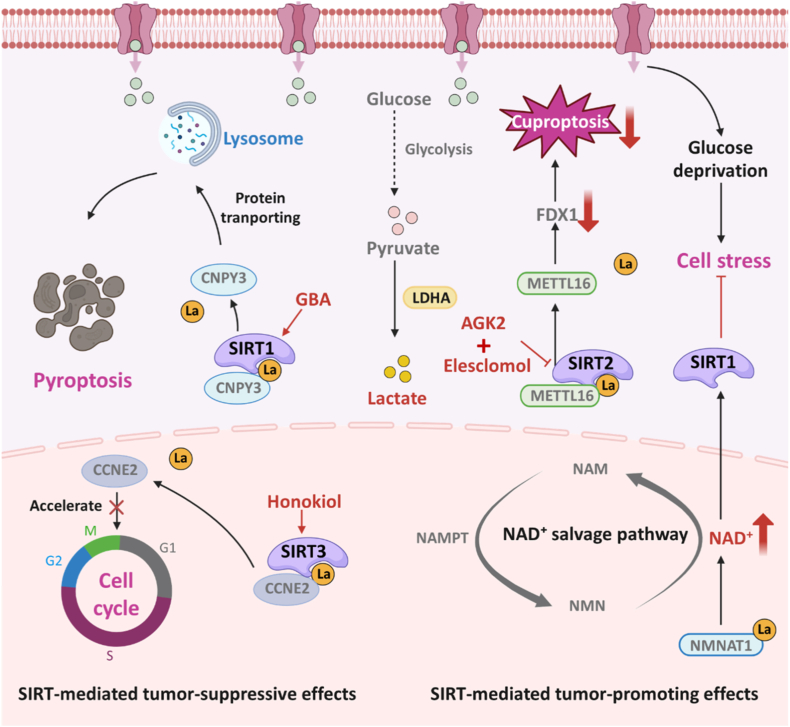
Schematic illustration of the context-dependent roles of Sirtuin-mediated delactylation in cancer, showing both tumor-suppressive and tumor-promoting effects. Created in https://Biorender.com.

Members of the Sirtuin family, as NAD^+^-dependent deacetylases, play more complex and specialized roles in the removal of protein lactylation (Fig. 15). In prostate cancer, gambogic acid (GBA) activates SIRT1, promoting the delactylation of canopy FGF signaling regulator 3 (CNPY3), which alters its subcellular localization, resulting in lysosomal rupture and tumor cell pyroptosis [100]. In hepatocellular carcinoma, activation of SIRT3 by honokiol enhances cyclin E2 (CCNE2) delactylation and inhibits tumor growth [101].

However, the function of Sirtuins-based delactylation is inconsistent, it could also help tumor cells in acclimatizing to cellular stress and evading treatment. In gastric cancer, lactylation of METTL16 enhances its m6A methyltransferase activity, which stabilizes FDX1 mRNA through m6A modification, ultimately increasing FDX1 protein levels and inducing cuproptosis; in contrast, SIRT2-mediated delactylation of METTL16 reduces cuproptosis mediated by FDX1 and leads to resistance. Co-treatment with the copper ionophore elesclomol and the SIRT2 inhibitor AGK2 significantly reduces tumor cell viability and tumor volume in mice. Co-treatment with the copper ionophore elesclomol and the SIRT2 inhibitor AGK2 significantly reduces tumor cell viability and tumor volume in mice [102]. Additionally, in pancreatic cancer under glucose deprivation, lactate enhances nicotinamide mononucleotide adenylyltransferase 1 (NMNAT1) lactylation to sustain the nuclear NAD^+^ salvage pathway. Elevated NAD^+^ levels further activate SIRT1, suppressing stress signaling, and supporting tumor cell survival [117]. These findings highlight that modulation of delactylation may require target-specific strategies depending on the cellular context.

## 4 Conclusion

Owing to even in normoxic environments, malignant tumor cells use the Warburg effect to thrive and preferentially use glycolysis over mitochondrial oxidative OXPHOS to produce energy, lactate accumulation is a hallmark of tumor metabolic reprogramming. Moreover, tumor cells export lactate into the tumor microenvironment via MCT4, further acidifying TME and significantly impairing the antitumor functions of T cells, macrophages, and natural killer cells (NK cells), leading to the limited therapeutic efficiency of various treatments. Notably, lactate also serves as a precursor for protein lactylation, which activates and enhances the function of pro-oncogenic genes (Fig. 1). Thus, targeting tumor lactate metabolism and lactylation modification holds significant potential for improving current anticancer therapies (Fig. 2). In this review, we summarize recent advances in functional nanomaterials that modulate tumor lactate metabolism to enhance antitumor efficacy, including (1) inhibiting lactate production by delivering drugs or siRNA to selectively inhibit the function of key enzymes in glycolysis, such as HK2, PKM2, and LDHA; (2) blocking lactate efflux by inhibiting MCT4 through specific inhibitors and nanoparticles with galactose moieties on the surface to competitively bind to glycosylation sites on MCT4; and (3) promoting lactate consumption via LOX-mediated oxidation of lactate, carbonate-containing nanomaterial-based neutralization of lactate, and engineered bacterial-based metabolism of lactate. We also discuss current strategies for regulating lactylation, such as (1) suppressing LDHA and PKM2 function to disrupt lactate-driven epigenetic modifications, (2) blocking the enzymes in lactyl-CoA-dependent and -independent lactylation pathways to inhibit lactylation formation, and (3) enhancing the activity of delactylases to mitigate lactylation.

The diverse methodologies for modulating lactate metabolism and lactylation in tumors present specific advantages and drawbacks, which can be described as follows: the (1) The reduction of lactate formation, by targeting HK2, PKM2, or LDHA, exhibits significant metabolic selectivity and can significantly diminish glycolytic flow. Nonetheless, these strategies are susceptible to metabolic compensation, as tumor cells can swiftly transition to other energy routes, such as OXPHOS or glutamine metabolism, hence constraining their long-term effectiveness. (2) Suppressing MCT4 and MCT1 to limit lactate shuttling can directly disturb intracellular pH equilibrium within the tumor ecosystem, generating stress in tumor cells and simultaneously restructuring the immunosuppressive microenvironment. However, the effectiveness of this method is significantly contingent upon geographic accessibility within tumor areas, presenting hurdles for delivery efficiency and selectivity. (3) Strategies that promote lactate consumption or neutralization, such as LOX-based catalytic systems, alkaline nanomaterials, and engineered bacteria, reduce extracellular acidity and restore immune function, showing strong potential for tumor microenvironment remodeling. Notwithstanding these benefits, their utilization is constrained by oxygen reliance, catalytic longevity, biosafety, and controllability, especially in heterogeneous solid tumors. (4) Emerging strategies that directly regulate protein lactylation aim to decouple metabolism from lactylation signaling by targeting lactyltransferases or delactylases, providing a more precise approach for epigenetic intervention. This subject, while conceptually innovative, remains hindered by an unclear mechanistic knowledge, restricted targeting methods, and possible off-target consequences, complicating clinical application.

Regulating lactate metabolism and protein lactylation by targeting metabolic reprogramming and epigenetic modifications in the TME can significantly enhance the efficacy of various anticancer therapies, including (1) overcoming chemotherapy resistance. HK2 inhibitors, like LND, could disrupt the mitochondrial OXPHOS for sensitizing cancer cells to cisplatin. Additionally, PKM2 inhibition could sensitize the cancer cells for TMZ- and sunitinib-induced chemotherapy. Once CaCO_3_ neutralized the acidity of the TME, it increased the uptake of DOX by enhancing membrane permeability. Hypoxia-inducible expression of LOX in engineered *E. coli* enabled lactate depletion, H_2_O_2_ generation, and O_2_ depletion, which could enhance the therapeutic efficacy of hypoxia-sensitive drug TPZ. In cisplatin-resistant gastric cancer cells, stiripentol induced a significant reduction in lactate production and a decrease in lactylation at lysine 388 of the NBS1 protein by selectively inhibiting the LDHA activity, which could markedly suppress tumor growth and prolong survival in mouse models when used in combination with cisplatin. Moreover, stiripentol offers potential to overcome TMZ resistance in glioblastoma through reduced histone H3K9 lactylation and downregulated LUC7L2-mediated intron retention in MLH1. (2) enhancing immunotherapy. HK2 and LDHA inhibition by LND and GSK drugs could enhance the CD8^+^ T cell cytotoxicity by reprogramming TME with reduced lactate generation. LOX-catalyzed lactate oxidation and H_2_O_2_ generation can further react with L-Arg to produce NO for vascular normalization and immune suppression alleviation. During the lactate neutralization process of NaHCO_3_ nanoparticles, the released high level of sodium ions could increase intracellular osmotic pressure for pyroptosis and ICD, thereby promoting the release of DAMPs and pro-inflammatory cytokines to enhance the antitumor immune response. Based on the triple-combination strategy of CaCO_3_ neutralizeing intratumoral lactate, CAT decomposing H_2_O_2_ into O_2_ to alleviate hypoxia, and anti-PD-1 restoring immune surveillance, the CAR-T efficacy could achieve complete tumor remission in 80% of mice. (3) sensitizing other strategies. HK2 inhibition could suppress lactate production and enhance tumor cell death induced by photothermal treatment. LDHA gene knockout could enhance the Cu^2+^-induced cuproptosis by reprogramming cellular metabolism. Blocking MCT4 stops lactate from leaving hypoxic tumor cells, causing lactate to build up inside, which leads to acidity and stress and catalyzes the Cu^+^/Cu^2+^ and Co^2+^/Co^3+^-based Fenton-like reaction for enhanced ferroptosis in tumor cells. Shikonin-induced significant reduction of PKM2 levels in BEAS-2B and NCI-H1395 cells could decrease H3K18la levels of AIM2 for sensitive ferroptosis. Additionally, D34-919, identified through small-molecule library screening, inhibits PKM2 tetramerization, reduces lactate production, and decreases XRCC1 lactylation, thereby suppressing chemoradiotherapy resistance in GBM.

Despite promising advances in targeting tumor lactate metabolism and lactylation for modulating the tumor microenvironment, overcoming therapy resistance, and improving immunotherapy, several key challenges remain: (1) The dynamic metabolic plasticity of tumor cells represents a central obstacle to lactate-targeted therapies. Upon inhibition of a single pathway, cancer cells rapidly activate compensatory metabolic reprogramming: oxidative cells deprived of lactate import may switch to glycolysis and compete for glucose, while deeply hypoxic cells may initiate autophagy or utilize glutaminolysis to survive [118]. Moreover, metabolic adaptation is often accompanied by epigenetic remodeling, limiting the durability of targeted interventions [119]. Although nanoscale co-delivery strategies have partially mitigated this plasticity, complete blockade of metabolic escape remains elusive. (2) The field of lactylation modulation faces fundamental mechanistic and translational hurdles. First, the mapping of lactylation in tumors is incomplete, and the dynamic regulatory network, encompassing the spatiotemporal interactions between lactate flow and delactylases like the Sirtuin family, is still debated. Second, causal links between specific lactylation sites and immunosuppressive pathways are poorly defined. Third, the absence of precise targeting instruments limits the efficacy of interventions. While current inhibitors, such as stiripentol for LDHA, can diminish lactate production, they fail to specifically inhibit lactylation, and small compounds aimed at lactyltransferases carry off-target dangers. (3) Clinical translation of nanomaterials targeting lactate metabolism encounters multiple barriers. First, biosafety is a paramount problem, since the prolonged presence of non-degradable nanomaterials may induce chronic inflammation or fibrosis [120], and the toxicity profiles of their metabolites require more rigorous evaluation using advanced *in vitro* and *in vivo* models. Second, delivery efficiency warrants attention, as abnormal vasculature and dense TME in solid tumors impede deep penetration, while metabolic variability hinders accurate targeting (e.g., MCT4 inhibitors must access hypoxic regions). Third, complex multi-functional nano systems (e.g., co-loaded LOX/chemotherapy platforms) suffer from batch-to-batch variability in properties like size and drug loading, presenting significant challenges for large-scale material fabrication.

To advance the clinical translation of lactate-targeting antitumor therapies, several promising strategies emerge: (1) Multi-target synergistic intervention represents a core direction to counteract metabolic plasticity. Smart nanocarriers capable of co-delivering lactate metabolism inhibitors, therapeutic agents, and lactylation modulators can enable coordinated metabolic and epigenetic blockade, disrupting the compensatory networks of cancer cells. In particular, the incorporation of protein lactylation–directed agents, such as site-specific cell-penetrating peptides or regulators of lactylation-driven signaling effectors, may further enhance therapeutic precision by selectively interrupting key oncogenic lactylation rather than globally suppressing metabolism. (2) Future efforts should focus on spatiotemporally controlled nano-delivery systems for silencing lactyltransferase gene clusters and simultaneously blocking both lactyl-CoA-dependent and -independent pathways to realize multidimensional targeting of protein lactylation. Currently, nanomaterials that directly regulate key enzymes involved in lactylation remain largely unexplored. In contrast, several emerging strategies have focused on intervening in downstream signaling pathways driven by lactylation, which represents an effective indirect means to modulate lactylation-related biological outcomes. For example, in the tumor microenvironment, lactate accumulation-induced H3K18la significantly promotes the transcription of the HECT domain E3 ubiquitin protein ligase 2 (HECTD2), thereby contributing to lenvatinib resistance in hepatocellular carcinoma. However, a PLGA-PEG-based nanoparticle delivery system with loading of siRNA against HECTD2 could specifically suppress the HECTD2 expression both *in vitro* and *in vivo*, ultimately reversing the drug-resistant phenotype mediated by the H3K18la–HECTD2 signaling axis [121]. (3) Materials innovation and delivery optimization are essential to overcome translational barriers. The development of biodegradable smart nanocarriers that co-deliver multi-target inhibitors (both metabolic and epigenetic) and feature biomimetic membranes enhances tumor penetration. Additionally, acid- and enzyme-dual-responsive release mechanisms can improve specificity and efficacy. Advanced nanoplatforms that integrate controlled intracellular delivery, lactylation-responsive release, and minimal off-target interference are particularly advantageous for translating lactylation-targeted strategies into clinically viable therapies. Lactate-sensitive biosensors may allow real-time monitoring of metabolic reprogramming effects, while machine learning can assist in customizing individualized dosage regimens. Ultimately, the integration of mechanism-driven research, improved materials, and clinical insights will enable the translation of lactate-targeted therapeutics from laboratory to clinical use for enhanced precision oncology.

## CRediT authorship contribution statement

**Shuzhe Cai:** Writing – original draft, Writing – review & editing. **Siqi Li:** Writing – original draft, Writing – review & editing. **Jingjing Liu:** Funding acquisition, Investigation, Writing – original draft, Writing – review & editing.

## Declaration of competing interest

The authors declare that they have no known competing financial interests or personal relationships that could have appeared to influence the work reported in this paper.

## Data Availability

Data will be made available on request.
